# Expression patterns of *semaphorin7A *and *plexinC1 *during rat neural development suggest roles in axon guidance and neuronal migration

**DOI:** 10.1186/1471-213X-7-98

**Published:** 2007-08-29

**Authors:** R Jeroen Pasterkamp, Sharon M Kolk, Anita JCGM Hellemons, Alex L Kolodkin

**Affiliations:** 1 Solomon H. Snyder Department of Neuroscience and Howard Hughes Medical Institute, The Johns Hopkins University School of Medicine, 1001 PCTB, 725 North Wolfe Street, Baltimore, MD 21205, USA; 2 Department of Pharmacology and Anatomy, Rudolf Magnus Institute of Neuroscience, University Medical Center Utrecht, Universiteitsweg 100, 3584 CG, Utrecht, The Netherlands

## Abstract

**Background:**

Although originally identified as embryonic axon guidance cues, semaphorins are now known to regulate multiple, distinct, processes crucial for neuronal network formation including axon growth and branching, dendritic morphology, and neuronal migration. Semaphorin7A (Sema7A), the only glycosylphosphatidylinositol-anchored semaphorin, promotes axon growth in vitro and is required for the proper growth of the mouse lateral olfactory tract in vivo. Sema7A has been postulated to signal through two unrelated receptors, an RGD-dependent α1β1-integrin and a member of the plexin family, plexinC1. β1-integrins underlie Sema7A-mediated axon growth and Sema7A function in the immune system. Sema7A-plexinC1 interactions have also been implicated in immune system function, but the neuronal role of this ligand-receptor pair remains to be explored. To gain further insight into the function(s) of Sema7A and plexinC1 during neural development, we present here a detailed analysis of *Sema7A *and *plexinC1 *expression in the developing rat nervous system.

**Results:**

In situ hybridization revealed select expression of *Sema7A *and *plexinC1 *in multiple neuronal systems including: the olfactory system, the hypothalamo-hypophysial system, the hippocampus, the meso-diencephalic dopamine system, and the spinal cord. Within these systems, *Sema7A *and *plexinC1 *are often expressed in specific neuronal subsets. In general, *Sema7A *transcript levels increase significantly towards adulthood, whereas *plexinC1 *expression decreases as development proceeds.

*PlexinC1*, but not *Sema7A*, is strongly expressed by distinct populations of migrating neurons. In addition to neuronal expression, *Sema7A *and *plexinC1 *transcripts were detected in oligodendrocytes and ependymal cells, respectively.

**Conclusion:**

*Sema7A *and *plexinC1 *expression patterns are consistent with these proteins serving both cooperative and separate functions during neural development. The prominent expression of *plexinC1 *in several distinct populations of migrating neurons suggests a novel role for this plexin family member in neuronal migration.

## Background

The formation of neural circuits during development depends on a precise series of molecular and cellular events. Once neurons have migrated to their final destination, they elaborate axons and dendrites along predetermined routes in the developing embryo to establish highly specific connections with their targets. Semaphorins, a large family of secreted and membrane-associated proteins, are instrumental in establishing patterns of neuronal connectivity and influence many different aspects of neuronal network formation including axonal and dendritic growth, branching, guidance and pruning, target recognition, and synapse formation [1]. Significant progress has been made in understanding how semaphorins provide guidance for extending neurites. Their contribution to other aspects of neuronal network formation is, however, less well understood. For example, in addition to their chemotropic effects, semaphorins can exert neurite growth promoting effects [1]. For example, both in vitro and in vivo semaphorin7A (Sema7A, also known as CDw108) functions as an axon growth-promoting factor [2]. Sema7A is the only glycosylphosphatidylinositol (GPI)-anchored semaphorin described to date, and it was first identified as a member of the semaphorin family in a search for vertebrate orthologues of class 8 viral semaphorins [3,4]. In addition to promoting neurite outgrowth, Sema7A expression and function studies support it playing a role in immune system function and bone morphogenesis. In the immune system, Sema7A is expressed in the lymphoid and myeloid lineages and is known to affect several immunological functions including immune cell proliferation, chemotaxis and cytokine release [5]. Furthermore, Sema7A defines the John-Milton-Hagen human blood group on erythrocytes, which is implicated in the pathogenesis of a clinically benign autoimmune disorder [6]. A role for Sema7A in bone formation is supported by the observation that Sema7A can regulate osteoclast differentiation and pre-osteoblastic cell migration in vitro [7]. In line with this observation, polymorphisms in the human *SEMA7A *gene were recently found to be associated with bone mineral density and fracture risk in postmenopausal women [8].

Studies of *Sema7A *expression and function in chick and mouse embryos define multiple, distinct, roles for Sema7A in the developing nervous system. *Sema7A *expression during early chick embryonic development hints at its involvement in neural crest cell migration and/or differentiation [9]. At later developmental stages, when neuronal connections are being established and remodelled, Sema7A promotes growth and branching of certain neurite subsets [2,10]. For example, Sema7A enhances the neurite outgrowth of cultured cortical, olfactory and sensory, but not vomeronasal, neurons. In support of these observations, *Sema7A*^-/- ^mice show marked defects in the growth of olfactory bulb axons projecting through the lateral olfactory tract during development [2].

Members of the plexin family are the major semaphorin receptors in the nervous system and Sema7A has been shown to bind to one of the nine vertebrate plexins, plexinC1, in vitro [11]. Surprisingly, the axon growth promoting effects of Sema7A do not require plexinC1 but instead depend on β1-integrins and associated signalling cascades [2]. However, the ability of Sema7A to bind plexinC1, the implication of Sema7A-plexinC1 signaling in immune system function, and the expression of the genes encoding both proteins at times when select neuronal connections are established [2,4,11-15], suggests that Sema7A-plexinC1 interactions participate in neuronal network formation. Unfortunately, the distribution of Sema7A and plexinC1 during late embryonic and postnatal neural development, when important events in neuronal network formation and remodelling occur, is not well characterized. As a step toward obtaining a better understanding of the separate and cooperative functions of Sema7A and plexinC1 during neural development, we have determined the spatiotemporal expression patterns of *Sema7A *and *plexinC1 *in the developing and adult rat nervous system.

## Results

### *Sema7A *and *plexinC1 *are differentially expressed during neural development

**Figure 1:**
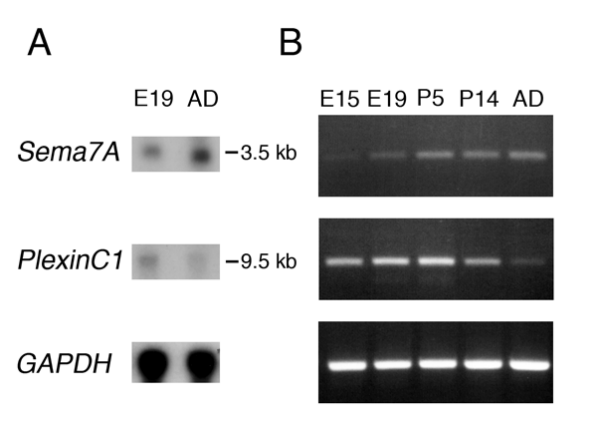
***Sema7A *and *plexinC1 *are differentially expressed during rat neural development**. Expression of *Sema7A *and *plexinC1 *during rat neural development was analyzed by Northern blot analysis (A) and RT-PCR (B). (A) Northern blot analysis of 40 μg E19 or adult (AD) rat whole-brain RNA was performed using *Sema7A*-specific probes. The blot was reprobed twice, first for *plexinC1*, and subsequently for *GAPDH *to control for the amount of RNA loaded in each lane. (B) E15 to adult rat whole-brain RNA was isolated and used for semiquantitative RT-PCR using *Sema7A*-, *plexinC1*- and *GAPDH*-specific primers. Experiments were done at least three times with similar results.

In order to gain further insight into how Sema7A and plexinC1 function in the developing nervous system, we assessed in detail the spatiotemporal patterns of *Sema7A *and *plexinC1 *expression during rat neural development. First, we examined overall expression of *Sema7A *and *plexinC1 *in the embryonic and adult brain by using Northern blot and reverse transcription (RT)-PCR analyses. Northern analysis of whole-brain mRNA isolated from embryonic day (E)19 or adult rats revealed a single *Sema7A *transcript of approximately 3.5 kilobase pairs (kb) and a single *plexinC1 *transcript of ~9.5 kb. These sizes are consistent with those reported previously [2,16,17]. The *Sema7A *Northern RNA band was stronger in the adult brain compared to E19. In contrast, *plexinC1 *expression is somewhat stronger in embryonic neural tissue (Fig. 1A). To analyze these differential temporal expression profiles in more detail, we performed RT-PCR using *Sema7A*- and *plexinC1*-specific primers. Consistent with our Northern results, we found *Sema7A *weakly expressed at E15 and its expression markedly increased towards adulthood. Conversely, *plexinC1 *signals were strongest during late embryonic and early postnatal development, decreasing towards adulthood (Fig. 1B). Overall, these results reveal striking complementary temporal expression profiles for *Sema7A *and *plexinC1 *during rat neural development.

To further determine the spatial and temporal distributions of *Sema7A *and *plexinC1*, we performed in situ hybridization analyses on tissue sections of the embryonic, postnatal and mature rat nervous system. Staining was absent from control sections, which were processed with sense probes [see Additional file 1]. In support of our Northern and RT-PCR data (Fig. 1), in situ hybridization revealed that *Sema7A *levels increase towards adulthood, whereas *plexinC1 *signals generally decline. For our expression studies, we largely focused on the olfactory system, hypothalamus and pituitary, hippocampus, meso-diencephalic dopamine (mdDA) system, and spinal cord, all of which display distinguishing features of *Sema7A *and *plexinC1 *expression.

### *PlexinC1 *and *Sema7A *expression in the hypothalamo-hypophysial system

**Figure 2:**
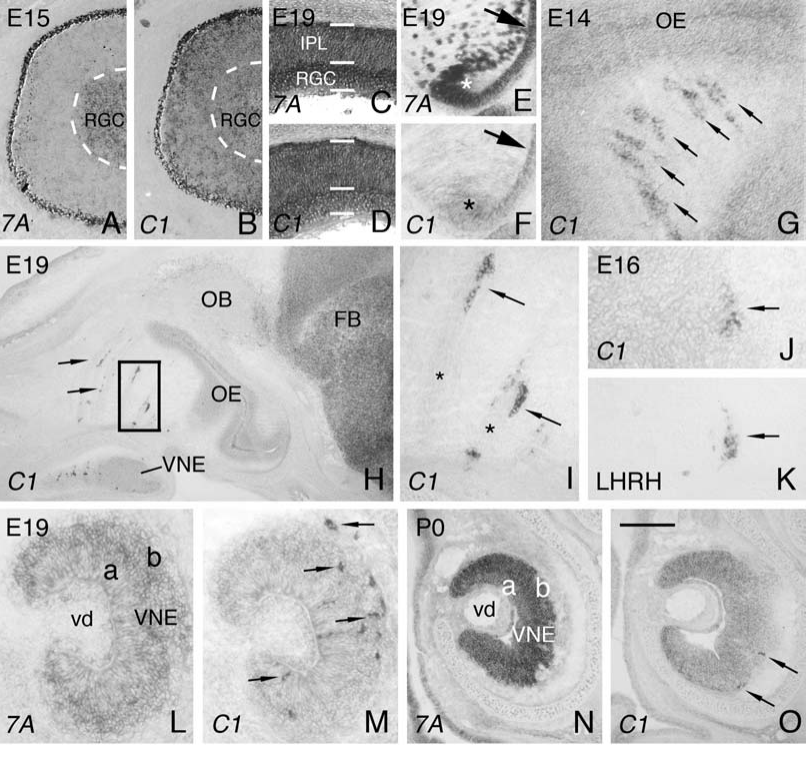
***PlexinC1 *is expressed in migrating LHRH neurons**. In situ hybridization for *Sema7A *and *plexinC1 *on consecutive horizontal (A-F), sagittal (G-K) and coronal (L-O) sections of the developing eye and nasal region. Rostral is to the top in A-F and to the left in G-K. Dorsal is to the top in L-O. (A, B) At E15, *Sema7A *and *plexinC1 *are expressed in the retinal ganglion cell layer (RGC). White line indicates the border of the RGC. (C, D) At E19, both *Sema7A *and *plexinC1 *are expressed in the inner plexiform layer (IPL) and RGC. (E, F) In addition, *Sema7A *and *plexinC1 *are expressed in the lens (arrow) and the lens epithelium (asterisk). (G-I) In the nasal region, *plexinC1 *is expressed in clusters of cells (arrows in G-I) located in close proximity to vomeronasal axon fascicles (asterisks in I). Panel I shows a higher magnification of the boxed area in panel H. (J, K) In situ hybridization for *plexinC1 *and immunohistochemistry for luteinizing hormone-releasing hormone (LHRH) on consecutive sections reveal that *plexinC1 *is expressed in migrating LHRH neurons. Arrow indicates a cell cluster that expresses *plexinC1 *and LHRH. (L, M) At E19, numerous *plexinC1*-positive LHRH neurons can be detected within and immediately outside the vomeronasal epithelium (VNE; arrows in M). (N, O) At P0, the majority of LHRH neurons have migrated to the forebrain and only few *plexinC1*-positive LHRH neurons remain in the VNE (arrows in O). (L-O) Note that both *Sema7A *and *plexinC1 *are expressed by vomeronasal neurons in the apical (a) and basal (b) VNE. 7A, Sema7A; C1, plexinC1; FB, forebrain; OB, olfactory bulb; OE, olfactory epithelium; vd, vomeronasal duct. Scale bar 320 μm (A, B), 85 μm (C, D), 95 μm (E, F), 90 μm (G), 675 (H), 25 μm (I), 80 μm (J, K), 150 μm (L, M), and 275 μm (N, O).

In the E15 and E19 eye, *Sema7A *and *plexinC1 *are expressed in the retinal ganglion cell (RGC) layer (Fig. 2A–D). *Sema7A *and *plexinC1 *are also detected in the inner plexiform layer (IPL) at E19 (Fig. 2C, D). In addition, *Sema7A *and *plexinC1 *are expressed in the lens and the lens epithelium (Fig. 2E, F). Analysis of the nasal region, revealed a population of *plexinC1*-expressing cells in close proximity to axon fascicles emerging from the vomeronasal organ (VNE) (Fig. 2G–I). These cells are organized in larger clusters or cords, reminiscent of migrating luteinizing hormone-releasing hormone (LHRH) neurons [18,19]. In rodents, LHRH neurons originate in the olfactory placode, migrate across the nasal septum along vomeronasal nerve branches and enter the forebrain arching into the septal-preoptic area and hypothalamus [20]. Indeed, in situ hybridization for *plexinC1 *and immunohistochemistry for LHRH on consecutive sections confirmed that *plexinC1*-positive neurons in the nasal compartment express LHRH (Fig. 2J, K). In contrast, *Sema7A *transcript was not found in migrating LHRH neurons (not shown). *PlexinC1*-positive LHRH cells are also present within the VNE. In addition, both *Sema7A *and *plexinC1 *are expressed in vomeronasal cells throughout the apical and basal VNE (Fig. 2H, L–O). As neural development progresses, the number of LHRH neurons in the nasal compartment decreases as a result of their migration into the forebrain [20].

**Figure 3:**
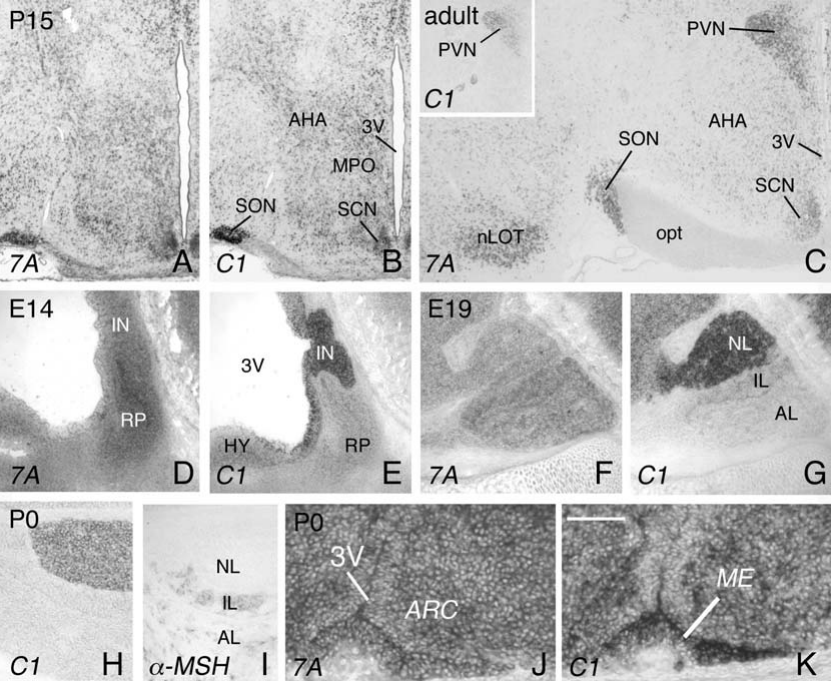
***Sema7A *and *plexinC1 *expression in the developing hypothalamus and pituitary**. In situ hybridization for *Sema7A *and *plexinC1 *on consecutive coronal (A-C, H-K) and sagittal (D-G) sections of the hypothalamus and pituitary. Dorsal is to the top. (A, B) *Sema7A *and *plexinC1 *are expressed in highly overlapping patterns during hypothalamic development including in the anterior hypothalamus (AHA) and medial preoptic area (MPO). (C) *Sema7A *is strongly expressed in several adult hypothalamic nuclei including the suprachiasmatic nucleus (SCN), paraventricular nucleus (PVN) and supraoptic nucleus (SON). In contrast, *plexinC1 *expression in the adult hypothalamus is largely restricted to the PVN (insert in panel C). (D, E) In the E14 pituitary, *Sema7A *expression is strongest in Rathke's pouch (RP), whereas *plexinC1 *labels the neural part of the pituitary (IN). (F, G) At E19, *Sema7A *is strongly expressed in the anterior (AL) and intermediate (IL) lobes of the pituitary and *plexinC1 *is largely confined to the neural lobe (NL). The weak *plexinC1 *signals observed in the intermediate lobe at E19 are not detected at later developmental stages (H). (H, I) In situ hybridization for *plexinC1 *and immunohistochemistry for α-melanophore-stimulating hormone (α-MSH), a marker of the intermediate and anterior pituitary, on consecutive sections. Note that *plexinC1 *is confined to the neural, i.e α-MSH-negative, part of the pituitary at P0. (J, K) At P0, *Sema7A *and *plexinC1 *are expressed in the projection area of LHRH axons, the median eminence (ME). *Sema7A *and *plexinC1 *expression can also be detected in the arcuate nucleus (ARC). 3 V, third ventricle; 7A, Sema7A; C1, plexinC1; HY, hypothalamus; IN, infundibulum; nLOT, nucleus of the lateral olfactory tract; opt, optic nerve. Scale bar 390 μm (A, B), 425 μm (C), 750 μm (insert C), 211 μm (D, E), 360 μm (F, G), 425 μm (H, I), and 40 μm (J, K).

This is consistent with our observation that the number of *plexinC1*-positive neurons along vomeronasal nerve branches in the nasal region and in the VNE decreases as development progresses (Fig. 2G–O). At P0, only a few *plexinC1*-positive LHRH neurons were detected in the VNE (Fig. 2O). Unfortunately, widespread *plexinC1 *expression in the forebrain prevented us from monitoring the intracerebral migration of the *plexinC1*-positive LHRH neurons (Fig. 2H). However, at later developmental stages *plexinC1*-expressing neurons are found in areas known to harbour LHRH neuron cell bodies, such as the medial preoptic area (MPO; Fig. 3B). Overall, these results suggest a novel role for plexinC1 in neuronal cell migration.

Once LHRH neurons reach their final destination in the CNS they project axons to the median eminence (ME), where LHRH is deposited into the hypothalamo-hypophysial portal system to stimulate LH release from the anterior pituitary [21]. During development, the ME strongly expresses *plexinC1 *and to a lesser extent *Sema7A *(Fig. 3J, K). In addition to the ME, other portions of the developing hypothalamic area, including the supraoptic nucleus (SON), display prominent *Sema7A *and *plexinC1 *expression (Fig. 3A, B). As hypothalamic development progresses, *plexinC1 *expression becomes largely confined to the paraventricular nucleus (PVN), whereas *Sema7A *transcripts remain widely distributed throughout the hypothalamus (Fig. 3C). In contrast to LHRH axons, which terminate at the ME, fibers of magnocellular PVN and SON neurons pass through the ME to innervate the neural lobe of the pituitary [22]. Similar to LHRH projections to the median eminence, both developing PVN and SON projection neurons along with their target structure, the neural lobe of the pituitary, display strong *plexinC1 *expression during development (Fig. 3B, E, G, H).

These observations are in line with a recent study showing that *plexinC1 *is expressed in magnocellular PVN and SON neurons at E12.5 and E15.5 [14].

The pituitary consists of three lobes; the neural lobe, the intermediate lobe, and the anterior lobe. The neural lobe derives from diencephalic tissue and the anterior and intermediate lobes form from an invagination of the oral ectoderm called Rathke's pouch [22]. During pituitary development, expression of *Sema7A *is most pronounced in Rathke's pouch, and at later developmental stages in the intermediate and anterior lobes (Fig. 3D, F). In contrast, *plexinC1 *is largely confined to the neural part of the pituitary, with only weak expression observed in the intermediate lobe at late embryonic, but not postnatal or adult, stages (Fig. 3E, G, H, I). Overall, these *Sema7A *and *plexinC1 *expression patterns are consistent with Sema7A and plexinC1 playing prominent roles in neuronal migration and network formation in the hypothalamo-hypophysial system.

### *Sema7A *and *plexinC1 *expression in the olfactory system

**Figure 4:**
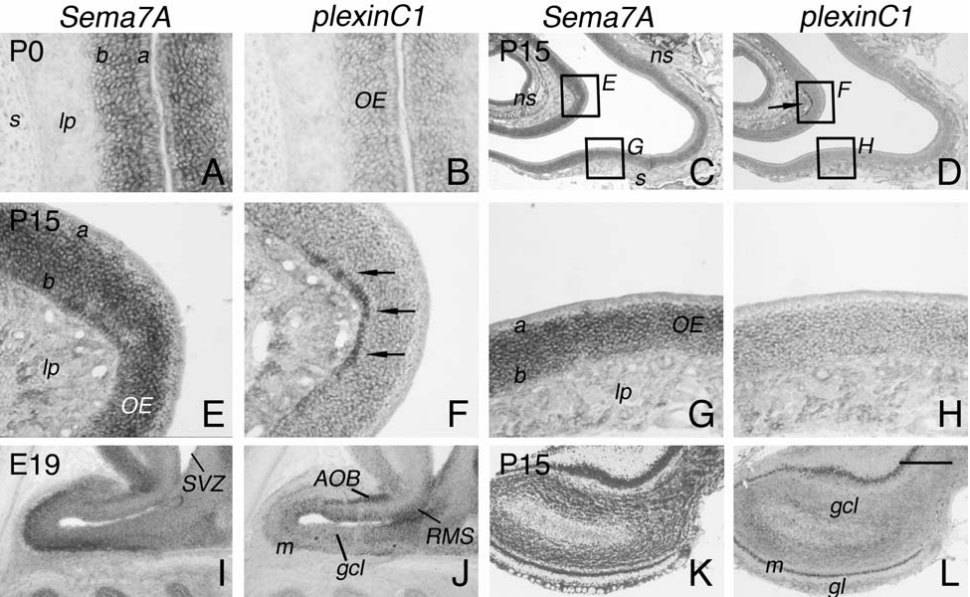
**Complementary patterns of *Sema7A *and *plexinC1 *expression in the olfactory system**. In situ hybridization for *Sema7A *and *plexinC1 *on consecutive horizontal (A-H) and sagittal (I-L) sections of the primary and accessory olfactory system. Caudal is to the top in A and B, and to the right in C-L. (A, B) During embryonic (not shown) and postnatal development, *Sema7A *and *plexinC1 *are expressed throughout the apical (a) and basal (b) layers of the olfactory epithelium (OE). No expression is found in the lamina propria (lp) and septum (s). (C-H) Panels E and F show P15 non-septal (ns) OE and represent higher magnifications of the boxed areas in C and D. Panels G and H show P15 septal OE and represent higher magnifications of the boxed areas in C and D. As postnatal development progresses, *Sema7A *signals in the OE increase whereas weak expression of *plexinC1 *is found throughout the apical (a) and basal (b) layers of the OE. (D, F) Note, however, the moderate-to-strong *plexinC1 *signals in cells in the basal part of a specific patch of OE (arrows in D and F). (I, J) At E19, *Sema7A *is expressed in the mitral (m) and granule cell (gcl) layers of the olfactory bulb (OB). In addition, low levels of *Sema7A *can be detected in the accessory olfactory bulb (AOB). *PlexinC1 *is expressed by a subset of granule and mitral cells. Strong *plexinC1 *expression is found in the AOB, rostral migratory stream (RMS) and subventricular zone (SVZ). (K, L) At P15, *Sema7A *and *plexinC1 *are expressed in mitral, tufted, granule and periglomerular cells. gl, glomerular layer. Scale bar 40 μm (A, B), 460 μm (C, D), 105 μm (E-H), 500 μm (I, J), and 775 μm (K, L).

In addition to the VNE (Fig. 2), expression of *Sema7A *and *plexinC1 *was observed in other portions of the primary and accessory olfactory systems. In the olfactory epithelium (OE), *Sema7A *was detected as early as E14, the earliest timepoint examined in this study, and expression levels dramatically increased towards adulthood. In postnatal and adult rats, cells in both the basal and apical parts of the epithelium displayed *Sema7A *signals (Fig. 4A, C, E, G). In contrast to *Sema7A*, expression of *plexinC1 *in the OE was weak at each developmental stage examined (Fig. 2G, H, 4B, D, F, H). One exception is a small population of cells in the most basal part of postnatal and adult OE that expresses *plexinC1 *at moderate-to-high levels. Interestingly, we detected only few of these *plexinC1*-positive cell patches scattered throughout non-septal OE (Fig. 4D, F, H). The target structure of OE neurons, the olfactory bulb (OB), also expresses *Sema7A *and *plexinC1*. Both gene products were detected in several distinct populations of OB neurons including mitral cells, tufted cells, granule cells, and periglomerular cells. However, whereas *Sema7A *was detected in the majority of OB neurons, *plexinC1 *was only present in a subset of mitral, tufted, granule and periglomerular cells from E16 to adult (Fig. 4I–L; not shown). Interestingly, *plexinC1 *transcripts were also found in different layers of the accessory olfactory bulb (AOB) and in cells in the rostral migratory stream (RMS) and subventricular zone (SVZ). Weak *Sema7A *expression was observed in the AOB but not in the RMS (4I, J). Furthermore, *Sema7A *and *plexinC1 *are present in the central nuclei in the brain that complete the primary and accessory olfactory pathways, including the piriform cortex and nucleus of the lateral olfactory tract (Fig. 3C).

### Hippocampal CA2 neurons express high levels of *Sema7A*

**Figure 5:**
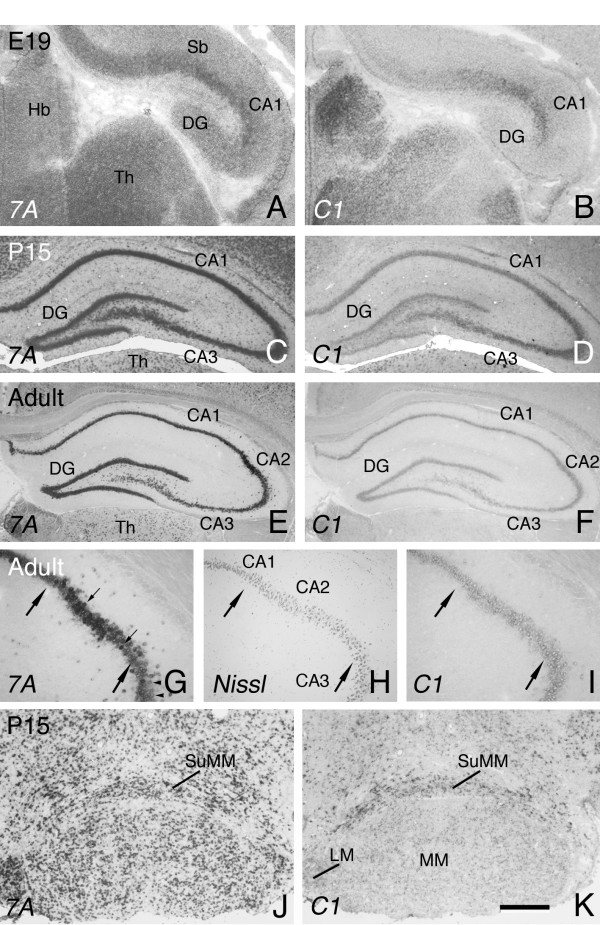
**Sema7A is enriched in CA2 hippocampal neurons**. In situ hybridization for *Sema7A *and *plexinC1 *on consecutive coronal sections of the hippocampus. Dorsal is to the top. (A, B) At E19, *Sema7A *is expressed in the dentate gyrus (DG) and CA1 region, whereas *plexinC1 *expression is restricted to the CA1 field. *Sema7A *and *plexinC1 *are also detected in the habenula (Hb) and thalamus (Th). (C-F) As development progresses, both *Sema7A *and *plexinC1 *can be detected in the DG, CA1 and CA3 fields. (E-I) Note that in the adult *Sema7A *signals are higher in the CA2 as compared to the adjacent CA1 and CA3 regions. Panels G and I are higher magnifications of the CA2 region in E and F, respectively. H shows a Nissl stain of the CA2 region (indicated by arrows in G-I) revealing its characteristic cellular organization. Small arrows in G indicate CA2 neurons, arrowheads indicate CA3 neurons. Note that CA2 neurons show a stronger *Sema7A *expression as compared to adjacent CA3 neurons. (J, K) CA2 neurons receive a particular prominent input from several subdivisions of the mammillary nucleus several of which display prominent *Sema7A *and *plexinC1 *labeling including the supramammillary nucleus (SuMM). 7A, Sema7A; C1, plexinC1; LM, lateral mammillary nucleus; MM, medial mammillary nucleus; Sb, subiculum. Scale bar 120 μm (A, B), 775 μm (C, D), 900 μm (E, F), 80 μm (G-I), and 420 μm (J, K).

At E19, both *Sema7A *and *plexinC1 *were detected in the CA1 region of the developing hippocampus, but only *Sema7A *was expressed in the dentate gyrus (DG), albeit at lower levels (Fig. 5A, B). As development progresses, both *Sema7A *and *plexinC1 *are found in the CA1-CA3 regions and in dentate granule and some hilar cells (Fig. 5C, D). In the adult, *Sema7A *and *plexinC1 *are expressed in all hippocampal fields (Fig. 5E, F). Although overall adult hippocampal expression of *plexinC1 *is somewhat lower than that at P15, the hippocampus is one of the few neural structures that display moderate-to-strong adult *plexinC1 *expression. Interestingly, *Sema7A *expression in pyramidal neurons of the CA2 field is markedly higher than in adjacent CA1 and CA3 neurons (Fig. 5E–I, 7G). The CA2 is a narrow field of large-sized pyramidal cells located distal to the end bulbs of the DG mossy fiber projection. Relatively little is known about the connections and function of neurons in the CA2 field. However, CA2 neurons have been reported to give rise to divergent intrahippocampal projections and to receive a particularly prominent input from several subdivisions of the mammillary nucleus, such as the supramammillary nucleus [23]. Interestingly, during both embryonic and postnatal development the supramammillary nucleus and also other parts of the mammillary body display prominent *Sema7A *and *plexinC1 *expression (Fig. 5J, K).

### *Sema7A *and *plexinC1 *define subsets of meso-diencephalic dopamine neurons

**Figure 6:**
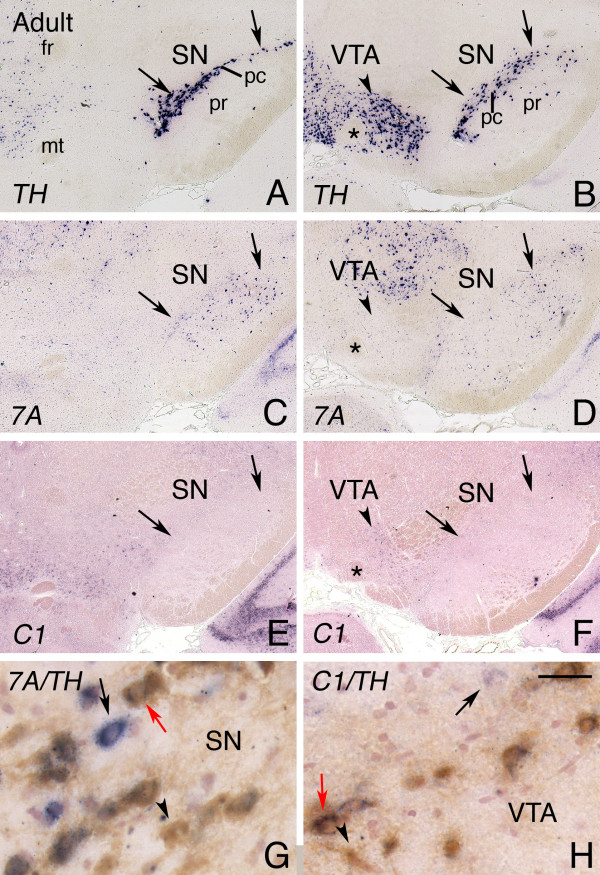
***S*ubsets of meso-diencephalic dopamine neurons express *Sema7A *and *plexinC1***. In situ hybridization for *tyrosine hydroxlyase *(*TH*), *Sema7A *and *plexinC1 *(A-F), and colocalization of TH protein and *Sema7A *(G) and of TH protein and *plexinC1 *(H) on coronal sections of the adult meso-diencephalic dopamine (mdDA) system. A, C and E show consecutive sections of the rostral part of mdDA system, B, D and F of its caudal part. Dorsal is to the top. (A, B) *TH *labels mdDA neurons in the subtantia nigra (SN) and ventral tegmental area (VTA). Arrows in A through F outline the position of the SN as visualized by TH labeling. Arrowheads in B, D and F indicate the central part of the VTA. (A-F) Note that whereas overlap in *TH *and *Sema7A *expression is largely confined to the lateral aspect of the SN (C, D), *TH *and *plexinC1 *predominantly overlap in the central part of the VTA (F, indicated by arrowhead). No *plexinC1 *expression is detected in the SN (E, F). Asterisk indicates the fasciculus retroflexus. (G, H) Colocalization of TH protein (brown) and *Sema7A *(G; purple) in the SN and of TH protein (brown) and *plexinC1 *(H; purple) in the VTA reveals *Sema7A *or *plexinC1*-positive mdDA neurons (red arrows in G, H), mdDA neurons that lack these transcripts (arrowheads in G, H), and non-mdDA neurons or cells that express *Sema7A *or *plexinC1 *(black arrows in G, H). 7A, Sema7A; C1, plexinC1; fr, fasciculus retroflexus; mt, mammillothalamic tract; pc, pars compacta; pr, pars reticulare. Scale bar 250 μm (A-F), and 65 μm (G, H).

Dopaminergic neurons in the substantia nigra pars compacta (SNc) and the ventral tegmental area (VTA) (i.e. meso-diencephalic dopamine (mdDA) neurons) play a fundamental role in movement coordination and several select behaviours, respectively. Within the SNc and VTA, mdDA neurons are organized in specific neuronal subsets each with unique molecular and functional properties [24]. Around E15, mdDA neurons, identified by the expression of *tyrosine hydroxylase *(*TH*), display moderate-to-strong *plexinC1 *expression. In contrast, no specific expression of *Sema7A *could be detected at this stage [see Additional file 1]. As development progresses, *Sema7A *signals in the mdDA system increase and expression of *plexinC1 *gradually decreases. In the adult, *Sema7A *and *plexinC1 *are found in non-overlapping subsets of mdDA neurons. Comparison of the *Sema7A *and *plexinC1 *expression patterns to that of *TH *reveal that a small number of mdDA neurons expressing *Sema7A *is located in the SNc, whereas *plexinC1*-positive mdDA neurons are predominantly found in the central part of the VTA (Fig. 6A–F). Double labelling, combining in situ hybridization for *Sema7A *or *plexinC1 *and immunohistochemistry for TH, reveals a small subset of neurons in the SNc and VTA that express TH and either *Sema7A *or *plexinC1*. In addition to these double-labelled neurons, cells expressing only *Sema7A*, *plexinC1 *or TH were detected. Thus, in addition to *Sema7A *or *plexinC1*-positive mdDA neurons, the SNc and VTA contain mdDA neurons that lack these transcripts and also non-mdDA neurons or cells that express *Sema7A *or *plexinC1 *(Fig. 6G, H).

**Figure 7:**
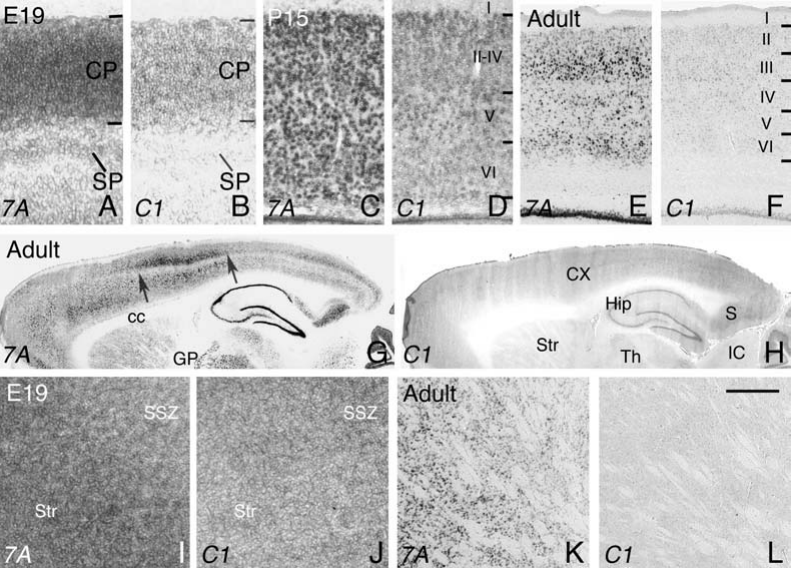
***Sema7A *and *plexinC1 *expression in the forebrain**. In situ hybridization for *Sema7A *and *plexinC1 *on consecutive coronal (A-F, I, J) and sagittal (G, H, K, L) sections of the cortex and striatum. Dorsal is to the top. (A, B) At E19, *Sema7A *and *plexinC1 *are expressed in the cortical plate (CP), whereas *Sema7A *is also expressed in the subplate (SP). In the postnatal (C, D) and adult cortex (E, F), *Sema7A *and *plexinC1 *can be found in layers II–VI. Note that *Sema7A *expression is highest in layers II and III in the adult (E). (G, H) Low magnification overview of *Sema7A *and *plexinC1 *expression in the adult brain. Note that cortical *Sema7A *expression is most pronounced within the barrel area of the presumptive somatosensory cortex (arrows indicate the anterior to posterior limits of the barrel cortex within layer V). In addition, *Sema7A *is strongly expressed in the subiculum (S), hippocampus (Hip), striatum (Str) and thalamus (Th). (I-L) *Sema7A *is expressed in a mediolateral gradient within the developing (I) and adult (J) striatum. Interestingly, *plexinC1 *is expressed in a lateromedial gradient in the embryonic (J) but not adult (L) striatum. 7A, Sema7A; C1, plexinC1; cc, corpus callosum; CX, cortex; GP, globus pallidus; IC, inferior colliculus; SSZ, striatal subventricular zone. Scale bar 140 μm (A, B), 120 μm (C, D), 2.2 mm (E, F), 17 mm (G, H), 180 μm (I, J), and 1.3 mm (K, L).

In addition to mdDA neurons themselves, the projection areas of these neurons also express *Sema7A *and *plexinC1*. SNc neurons predominantly innervate the dorsal striatum and caudate-putamen (the mesostriatal pathway), and VTA neurons innervate the ventral striatum and prefrontal cortex (the mesocorticolimbic system). During embryonic development, expression of both *Sema7A *and *plexinC1 *was detected in the cortical plate throughout the developing cortex (i.e. including the frontal cortex), whereas *Sema7A *was also expressed in the subplate region (7A, B). During postnatal development and in the adult, both *Sema7A *and *plexinC1 *transcripts were broadly distributed in cortical layers II to VI (Fig. 7C–H). Interestingly, cortical expression of *Sema7A *in the adult was most prominent in the barrel cortex (Fig. 7G). Elevated expression of Sema7A in the somatosensory cortex was only detected in the adult (not shown). *Sema7A *and *plexinC1 *are also expressed in the developing striatum. A mediolateral gradient of *Sema7A *expression was observed in the developing and mature striatum, whereas striatal *plexinC1 *expression was moderate-to-weak at embryonic stages and undetectable in the adult (Fig. 7I–L). Overall, these results show that *Sema7A *and *plexinC1 *not only define specific subsets of mdDA neurons, but also label their target structures.

### Differential expression of *Sema7A *and *plexinC1 *in the spinal cord, DRG and in muscle

**Figure 8:**
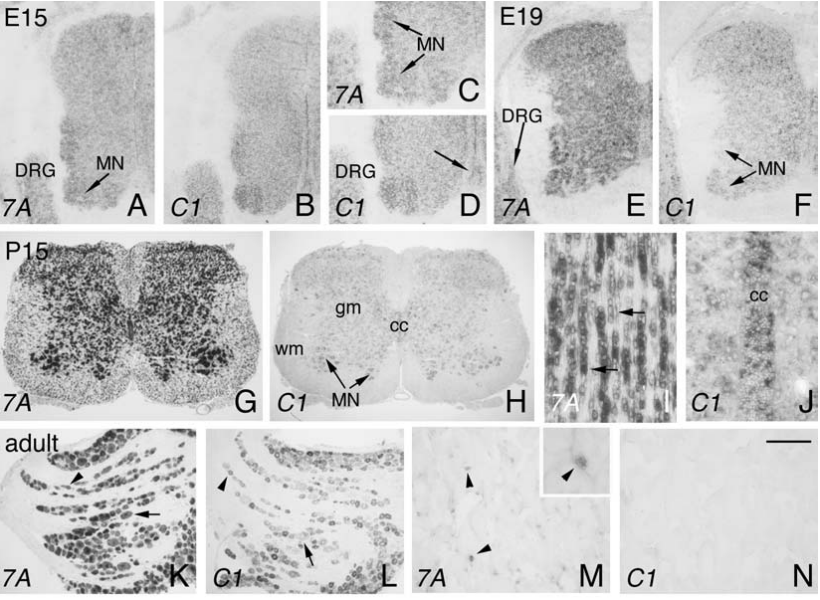
***Sema7A *and *plexinC1 *expression in spinal cord, DRG and muscle**. In situ hybridization for *Sema7A *and *plexinC1 *on consecutive coronal (A-H, K-N) and horizontal (I, J) sections of the spinal cord, dorsal root ganglion (DRG) and gastrocnemic muscle. (A, B) At E15, *Sema7A *and *plexinC1 *are detected in DRG neurons and throughout the spinal cord with highest expression in motor neurons (MN). (C, D) Panels C and D are higher magnifications of the ventral spinal cord shown in A and B, respectively. *PlexinC1 *is expressed in the floor plate (arrow in D). (E, F) Expression of *Sema7A *and *plexinC1 *in the E19 spinal cord and DRG. In the E19 DRG, most neurons express *Sema7A *but only few display *plexinC1 *signals. Both *Sema7A *and *plexinC1 *are expressed in motor neurons and in cells throughout the gray matter. (G, H) At P15, *Sema7A *and *plexinC1 *are expressed in motor neurons and throughout the gray matter (gm). No specific signals were detected in sections processed with corresponding sense controls. In addition, *Sema7A *is expressed in oligodendrocytes in the spinal cord white matter (arrows) (I) and *plexinC1 *labels ependymal cells lining the central canal (cc) (J). (K, L) In the adult, *Sema7A *and *plexinC1 *are expressed in adult small-(arrowhead) and large-diameter (arrow) DRG neurons. (M, N) *Sema7A *but not *plexinC1 *labels clusters of non-neuronal cells in the gastrocnemic muscle (arrowheads in M). The insert in M shows a higher magnification of a cluster of *Sema7A*-positive cells (indicated by an arrowhead). 7A, cc, central canal; Sema7A; C1, plexinC1; wm, white matter. Scale bar 150 μm (A, B), 90 μm (C, D), 225 μm (E, F), 540 μm (G, H), 125 μm (I, J), 220 μm (K, L), 500 μm (M, N), and 125 μm (insert in M).

We also analyzed *Sema7A *and *plexinC1 *expression in the spinal cord, starting at E15. *Sema7A *and *plexinC1 *were detected in dorsal root ganglion (DRG) neurons and throughout the spinal cord, with highest expression in motor neurons (Fig. 8A, B). In addition, *plexinC1*, but not *Sema7A*, is expressed in the floorplate, as observed previously [12](Fig. 8C, D). Similar expression patterns were observed at E19 (Fig. 8E, F). At P15 and in the adult, *Sema7A *and *plexinC1 *are expressed in motor neurons and throughout the grey matter of the spinal cord (Fig. 8G, H; not shown). In addition, *Sema7A *and *plexinC1 *were detected in non-neuronal cells in the postnatal and adult spinal cord. *Sema7A *is expressed by oligodendrocytes in the spinal cord white matter, as revealed by their characteristic organization in strings of small-sized cells (Fig. 8I). *PlexinC1 *is expressed in ependymal cells lining the central canal (Fig. 8J). In the adult DRG, both small- and large-diameter sensory neurons express *Sema7A*. *PlexinC1 *expression is most prominent in, but not restricted to, small-diameter DRG neurons (Fig. 8K, L). The gastrocnemic muscle is innervated by motor neurons located in the lumbar spinal cord. Interestingly, *Sema7A *is expressed in clusters of cells at the surface of individual muscle fibers, some of which closely resemble terminal Schwann cells (Fig. 8M, N). Overall, these results show that Sema7A and plexinC1 label subsets of (non)neuronal cells in spinal cord, DRG and muscle.

## Discussion

The GPI-linked semaphorin Sema7A was originally identified as a vertebrate homologue of a viral semaphorin [3,4]. Besides regulating aspects of immune system function and bone morphogenesis, Sema7A is a potent growth-stimulating factor for embryonic axons and postnatal dendrites [2,10]. Plexins are the predominant semaphorin receptors and, not surprisingly, Sema7A binds one of the nine vertebrate plexins, plexinC1 [11]. However, the axon growth-promoting effects of Sema7A do not rely on plexinC1 but instead require β1-integrins [2]. This is intriguing since *plexinC1 *and *Sema7A *transcripts are concomitantly expressed at the time neuronal connections are established and remodelled [2,4,12,13]. To better comprehend the neuronal function(s) of Sema7A and plexinC1 and to assess a potential role for neuronal Sema7A-plexinC1 signalling, we performed a detailed comparative analysis of *Sema7A *and *plexinC1 *expression patterns. The gene expression patterns reported here are consistent with both separate and cooperative Sema7A and plexinC1 functions.

### PlexinC1 is expressed in migrating neurons

In addition to their role as axonal repellents and attractants, semaphorins can also guide migrating cells through repulsive or attractive mechanisms. For example, secreted class 3 semaphorins (Sema3s) influence the migration of GABAergic interneurons from the medial ganglionic eminence and restrict the migration of trunk neural crest cells to the anterior sclerotome [25,26]. Similarly, class 6 transmembrane semaphorins (Sema6s) influence granule cell migration in the cerebellum and regulate the migration of endothelial and myocardial cells in the developing chick heart [27-29]. In many instances, these effects on migration require plexins. For example, plexinA1 mediates Sema6D-dependent migration events during cardiac morphogenesis and Sema3E-mediated somatic vascular patterning is dependent on plexinD1 [28,30]. In line with these observations, we find prominent *plexinC1 *expression in several classes of migratory neurons, including LHRH neurons in the nasal region, cells in the SVZ and RMS, and cells in the rhombic lip and external granule cell layer (EGL) [see Additional file 2]. These results suggest a novel role for plexinC1 in neuronal cell migration.

In the adult, LHRH regulates the release of anterior pituitary gonadotropes and is essential for reproduction. LHRH neurons originate from the embryonic nasal placode and migrate to the hypothalamus along olfactory/vomeronasal nerves. Several factors influence LHRH neuron migration, either directly or indirectly via the extension and guidance of vomeronasal axons, including growth factors (e.g. hepatocyte growth factor (HGF)) and axon guidance molecules (e.g. netrin-1) [20,31,32]. The *plexinC1 *expression patterns we report here are consistent with both direct and indirect effects on LHRH neuron migration. PlexinC1 could affect migrating LHRH neurons directly by mediating their selective adhesion to vomeronasal fibers or by influencing their subsequent migration. This would require LHRH cell-surface plexinC1 to bind membrane-associated proteins on vomeronasal axons. Interestingly, the only mammalian plexinC1 ligand identified to date, *Sema7A*, is expressed in embryonic and postnatal vomeronasal neurons. Alternatively, plexinC1 could act in a homophilic manner. This idea is supported by the ability of plexins to engage in homophilic interactions [33,34] and by the expression of *plexinC1 *in migrating LHRH neurons and vomeronasal neurons. A direct effect of plexinC1 on migrating neurons is further supported by recent work showing that plexinC1 is required for the proper allocation of developing magnocellular neurons to the PVN and SON, presumably by interacting with a repulsive ligand in the anterior hypothalamus [14]. Instead of affecting the migration of LHRH neurons directly, however, plexinC1 may influence vomeronasal/olfactory projections. The genetic ablation of guidance receptors such as DCC and neuropilin-2 results in severe defects in vomeronasal connectivity and as a result in misrouting of migrating LHRH neurons [35,36]. However, further work is needed to assess whether plexinC1, by analogy with other plexins, acts as a bona fide guidance or growth receptor. The finding that Sema7A does not affect vomeronasal axon growth and guidance in vitro [2] argues against a role for this potential plexinC1 ligand in the formation of vomeronasal nerve branches.

In addition to LHRH neurons, *plexinC1 *is expressed in SVZ/RMS cells and in the EGL of the developing cerebellum. The migration of neuroblasts in the RMS, and their subsequent differentiation into olfactory bulb granule and periglomerular cells is controlled by a complex molecular program which includes multiple growth and guidance molecules [37]. On basis of their expression in and around the SVZ, RMS, and throughout the olfactory system, semaphorins and their receptors likely contribute to the molecular control of RMS neuroblast migration and/or differentiation. The robust expression of *Sema7A *in the OB and in tissues surrounding the RMS is especially intriguing because of the expression of two distinct Sema7A (co)receptors in cells in the RMS: plexinC1 and β1 integrins [38]. β1 integrins are required for RMS cell chain formation and for the maintenance of glial tubes within the RMS. Although these morphological effects have been ascribed to distinct laminin isoforms, Sema7A shares at least one integrin receptor with laminins [39]. While the function of plexinC1 in the RMS remains to be addressed, cerebellar granule cells comprise another population of migrating neurons that expresses both *plexinC1 *and β1 integrins. Following their generation in the rhombic lip, cerebellar granule cell precursors migrate tangentially over the cerebellar plate to form the EGL. This initial migration is followed by a series of profound morphological changes and subsequent inward migration of postmitotic granule cells into the internal granular layer [40]. Inactivation of β1 integrins results in cerebellar granule cell precursor proliferation defects and in the disruption of cerebellar folia. These defects have been attributed to perturbed interactions between a sonic hedgehog-laminin protein complex and α6β1 or α7β1 integrins [41,42]. In immune cells α1β1, but not α6β1, mediates Sema7A function in monocyte activation, however, the role of α7β1 has not been addressed [39]. Instead of binding to β1 integrins on GCPs, however, Sema7A may bind plexinC1, which is enriched in cells in the rhombic lip and EGL. In addition to Sema7A, several other semaphorins are expressed in the developing cerebellum [27,43]. It is becoming clear that individual plexins can interact with members of different semaphorin classes. For example, plexinD1 can bind Sema3E and Sema4A, while plexinAs bind Sema6s and Sema3s, the latter via neuropilins [44,45]. Therefore, reassessing the semaphorin binding partners of plexinC1 may help to further define the role of this protein in neuronal cell migration and also in other biological processes.

### Sema7A and plexinC1 are expressed in specific subsets of neurons and glial cells

Neurons have often been grouped on basis of neurotransmitter expression or anatomical location. It is becoming increasingly clear, however, that within these chemically or anatomically defined neural systems distinct neuronal subsets exist, each displaying unique molecular and functional characteristics. For example, only a small subset of dopaminergic neurons in the SNc were recently found to express the netrin-1 receptor DCC and to specifically innervate the most dorsal aspect of the SNc projection area, the striatum [46]. As we report here, *Sema7A *and *plexinC1 *label subsets of neurons in several different systems including the olfactory system, cortex, hippocampus, mdDA system and neuromuscular system.

In the postnatal and adult hippocampus, *Sema7A *transcripts are enriched in the CA2 subfield. CA2 pyramidal neurons give rise to divergent intrahippocampal projections while receiving a prominent input from the mammillary nucleus [23]. *Sema7A*, *plexinC1 *and α1β1 integrins are expressed in the hippocampus and mammillary nucleus [47-49] suggesting that Sema7A signalling may influence the formation and maintenance of CA2 afferent and efferent projections. Another hallmark of CA2 pyramidal neurons is their relative resistance to cell death during temporal lobe epilepsy [50,51]. The observation that Sema7A acts as a survival factor for embryonic chick DRG neurons in vitro (R.J. Pasterkamp and A.L. Kolodkin, unpublished observations), together with its prominent expression in CA2 pyramidal neurons and epilepsy-induced (re-)expression of α1β1 integrins in hippocampal neurons and astrocytes [49,52], suggests that Sema7A might protect CA2 neurons against epileptic damage.

In the mdDA system *Sema7A *labels a subpopulation of SNc neurons, whereas *plexinC1 *is expressed by mdDA neurons in the central VTA. This observation is in line with a comparative analysis of gene expression profiles between the SN and VTA, reporting highest levels of *Sema7A *in mdDA neurons of the SN and of *plexinC1 *in the VTA [53]. Based on the expression of *Sema7A *and *plexinC1 *in the prefrontal cortex and striatum, it is tempting to speculate that these molecules contribute to the formation and maintenance of efferent and afferent dopaminergic midbrain connections.

### Is plexinC1 a Sema7A receptor?

Many semaphorin-mediated biological effects rely on receptor complexes that contain plexins as obligatory binding and/or signal transducing subunits. Sema7A binds plexinC1 [11], but thus far no functional role has been attributed to this interaction in neurons. Instead, the specific Sema7A functions in the neural and immune systems described to date require β1 integrins and not plexinC1 [2,39]. However, the ability of Sema7A and plexinC1 to physically interact with high affinity, together with their neuronal expression profiles, supports the notion that cooperative interactions between these proteins play some role during neural development. These expression data presented here show that plexinC1 frequently labels projection neurons, e.g. mdDA neurons in the VTA or sensory neurons in the OE, while the putative plexinC1 ligand Sema7A is present in their target structures, i.e. the prefrontal cortex and OB, respectively. While these observations are consistent with cooperative ligand-receptor interactions, it is evident that Sema7A and plexinC1 also function independently. For example, *Sema7A *is widely expressed in the adult nervous system, but *plexinC1 *expression is limited to only a few adult neural structures. In contrast, *plexinC1 *is expressed at high levels during early neural development, but mid-embryonic *Sema7A *expression is relatively weak. Overall, these observations indicate that additional interaction partners likely exist for Sema7A and plexinC1. Interestingly, α1 and β1 integrin subunits are expressed in the adult nervous system [48,54], while numerous other semaphorins, most of which have not yet been evaluated for plexinC1 binding, are present in the early embryonic nervous system (e.g. [55-57]). Furthermore, and in striking contrast to Sema7A, both plexinC1 and the β1 integrin subunit show a highly specific and spatially restricted expression patterns in the adult nervous system. This suggests that other Sema7A receptors may exist in addition to plexinC1 and β1 integrins.

## Conclusion

The *Sema7A *and *plexinC1 *expression patterns reported in this study are consistent with both cooperative and separate functions for the proteins encoded by these genes during neural development. Cooperative effects are supported by complementary expression patterns in various neuronal systems. Separate functions are strongly suggested at early and late stages of development, when either *plexinC1 *or *Sema7A *is prominently expressed. This suggests there are additional binding partners for plexinC1 and Sema7A. For plexinC1 these may include semaphorins belonging to other subclasses, and Sema7A already has been shown to signal through α1β1 integrins. The expression of *plexinC1 *in several groups of migratory neurons suggests a novel role for this plexin family member in cell migration. Future work will address these and other plexinC1 and Sema7A biological functions.

## Methods

### Animals

All animal procedures were conducted in strict compliance with approved institutional protocols. Timed-pregnant Wistar (Harlan CPB, Zeist, The Netherlands) were killed by intraperitoneal injection of a lethal dose of Euthesate (Ceva Sante Animale) and decapitated. Embryos were rapidly removed via cesarean section. The day of the vaginal plug was considered embryonic day (E)0.5. E14, E15, E16, E18, and E19 embryos were covered with Tissue-Tek (Sakura), and quickly frozen in dry ice-cooled 2-methylbutane (Sigma-Aldrich). At least five animals were processed for each stage. The day of birth was designated postnatal day (P)0. P0, P5, P14, and P15 rat pups and adult rats (2–4 months) were anesthetized and quickly decapitated. Neural tissues (brain, spinal cord, and dorsal root ganglia (DRG)) were dissected out and frozen in dry-ice cooled 2-methylbutane. Consecutive coronal, horizontal, and sagittal sections (10–20 μm) were cut on a cryostat and stored at -80°C until use.

### Northern blot analysis

Northern blot analysis was performed as described before [2]. In brief, RNA samples (40 μg) prepared from E19 and adult rat brain using TRIzol reagent (GibcoBRL) were size separated by electrophoresis in a 0.8% formaldehyde gel, transferred to a positively charged nylon membrane (Hybond-N^+^; Amersham Biosciences), and UV-crosslinked. DNA probes were labelled with [^32^P]-dCTP (Amersham Pharmacia Biotech) using the Prime-It II random labelling kit (Stratagene) and membranes were hybridized following standard procedures. A 362 base pair rat Sema7A cDNA probe was used to detect *Sema7A *[2]. Following detection of Sema7A, membranes were reprobed twice, first for plexinC1 using a 921 base pair cDNA probe (base pairs 3601–4522 of the mouse *plexinC1 *coding region), and then for GAPDH [2]. The *Sema7A *and *plexinC1 *probes do not cross-hybridize with the mRNA of other semaphorins and plexins, respectively.

### RT-PCR

Total RNA was isolated from E15, E19, P0, P14 or adult brain using TRIzol reagent (GibcoBRL). RT-PCR was performed as described before using primers specific for *Sema7A*, *plexinC1 *or *GAPDH*, as a control [58]. PCR conditions were as follows: 5 min/95°C, 30 s/95°C, 30 s/57°C, 45 s/72°C (27 cycles), 7 min/72°C. The following forward and reverse primers were used: mouse *Sema7A*; 5'-tgctggaacttggtgaatga-3' and 5'-atcttgcagccgattgaagt-3' (product size is 465 bp), mouse *plexinC1*; 5'-tggtggtgacgaggtacaaa-3' and 5'-tactgcactgctccatcagg-3' (468 bp), and rat *GAPDH*; 5'-ctcatgaccacagtccatgc-3' and 5'-atgtaggccatgaggtccac-3' (473 bp). The PCR products were run in an agarose gel and visualized with ethidium bromide. PCR amplification specificity was confirmed by sequencing.

### In situ hybridization

Although antibodies have been raised against mouse Sema7A and plexinC1 [16,17], these are not suitable for immunohistochemical detection of these proteins in analyses of the nervous system (R.J.P. and A.L.K., unpublished observations). Therefore we used in situ hybridization to study the distribution of *Sema7A *and *plexinC1 *in tissue sections. Non-radioactive in situ hybridization was performed using alkali-hydrolyzed digoxigenin-labelled cRNA probes transcribed from rat *Sema7A *(a 362 base pair fragment corresponding to nucleotides 364–726 of coding region) [2], mouse *plexinC1 *(a ~2.25 kb fragment encoded by EST AA940270) [2], or rat *tyrosine hydroxylase *(*TH*; basepair 915–1137 of the coding region) [59] cDNAs. The generation of cRNA probes and the in situ hybridization procedure were as described previously [58]. Sections subjected to the entire in situ hybridization procedure, but with no probe or sense probe added, exhibited no specific hybridization signal [see Additional file 1] [2]. The specificity of the in situ hybridization procedure was also inferred from the clearly distinct distribution patterns of *Sema7A*, *plexinC1 *and *TH*. In addition, we have performed experiments using a mouse *Sema7A *probe [4]. The expression patterns obtained with rat and mouse probes were identical.

### Immunohistochemistry

Immunohistochemistry was performed on cryosections as described previously [60]. In brief, sections were fixed for 1h at room temperature (RT), washed three times in Tris-buffered saline containing 0.2% TritonX-100 (TBS-T), blocked in TBS-T containing 0.2% bovine serum albumin (BSA) for 2h at RT, and incubated with primary antibodies overnight at 4°C. LHRH was detected with polyclonal rabbit antibodies (SW-1; 1:5000, a kind gift from Dr. S. Wray, National Institutes of Health, MD) [61] and α-MSH was detected with polyclonal rabbit antibodies (1:1000, a kind gift from Dr. F.W. van Leeuwen, University of Maastricht, The Netherlands) [62]. All primary antisera were diluted in TBS-T containing 0.2% BSA. No immunostaining was detected in control sections in which the primary antibodies were replaced by TBS-T. After three washes in TBS, sections were incubated with biotinylated goat anti-rabbit (1:100), all diluted in TBS-T containing 0.2% BSA, for 1 hr at RT. Then, sections were washed three times in TBS and incubated with avidin-biotin-peroxidase complex (Vectastain ABC kit; Vector Laboratories) in TBS containing 0.25% gelatin and 0.5% Triton X-100 for 1 hr at RT. After two washes in TBS and a brief wash in 50 mM Tris-HCl, pH 7.6, sections were reacted with a solution containing 0.035% DAB and 0.015% hydrogen peroxide in 50 mM Tris-HCl for 15 min at RT. The reaction was terminated by several washes in 50 mM Tris-HCl and sections were mounted in glycerol. Combined in situ hybridization for *Sema7A *or *plexinC1 *and immunohistochemistry for TH was performed as described previously [59] using polyclonal rabbit TH antibodies (1:1000, Pel-Freez).

## Authors' contributions

RJP carried out the experiments for figures 1, 2, 3, 4, 5 and 8 and performed data analyses. SMK and AJCGMH carried out the in situ hybridization and immunohistochemical experiments shown in figures 6 and 7. RJP and ALK conceived and coordinated the study, and RJP wrote the manuscript. All authors read and approved the final manuscript.

## Supplementary Material

Additional file 1 *Sema7A *and *plexinC1 *expression in the embryonic meso-diencephalic system. Description: In situ hybridization for *TH*, *Sema7A*, *plexinC1 *and sense control on consecutive coronal sections of the E15 meso-diencephalic dopamine (mdDA) system. (A) *TH *labels mdDA neurons. (A-C) Whereas no significant *Sema7A *expression is detected within the area of *TH *labelling, *plexinC1 *and *TH *expression clearly overlaps. (D) The sense control shows no specific labelling. Thus, at E15 *plexinC1 *but not *Sema7A *is expressed by mdDA neurons. Scale bar 60 μm (A-D). Click here for file

Additional file 2 *Sema7A *and *plexinC1 *expression in the embryonic and adult cerebellum. Description: In situ hybridization for *Sema7A *and *plexinC1 *on consecutive sagittal sections of the E19 (A, B) and adult cerebellum (C, D). (A, B) *Sema7A *is widely expressed throughout the developing cerebellum, whereas *plexinC1 *signals are enriched in the rhombic lip (arrow) and external granule cell layer (EGL). (C, D) In the adult cerebellum, *Sema7A *is expressed in Purkinje cells (P) and granule cells, whereas *plexinC1 *only labels few cells in the granule cell layer (GCL). Scale bar 150 μm (A, B), and 245 μm (C, D). Click here for file
