# Supplementary figures and images for: Expression patterns of semaphorin7A and plexinC1 during rat neural development suggest roles in axon guidance and neuronal migration
Source: BMC Dev Biol. 2007 Aug 29;7:98. doi: 10.1186/1471-213X-7-98 (PMC2008261; doi:10.1186/1471-213X-7-98)

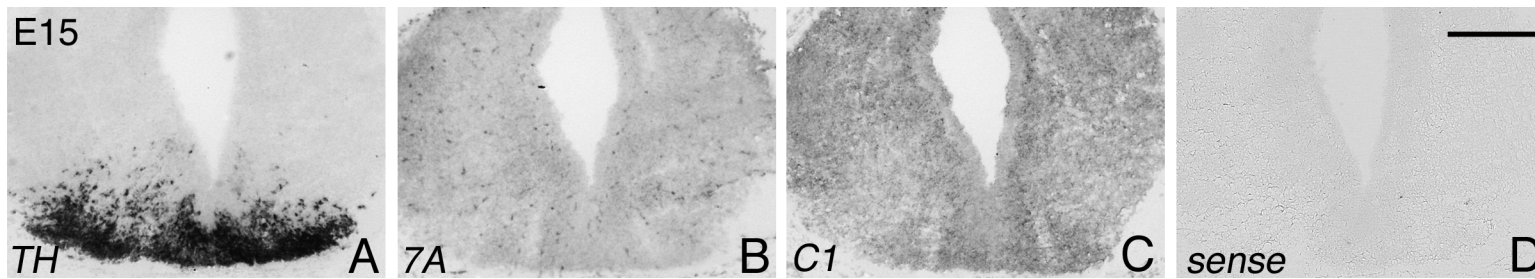

Supplement: Additional file 1 — Sema7A and plexinC1 expression in the embryonic meso-diencephalic system. Description: In situ hybridization for TH, Sema7A, plexinC1 and sense control on consecutive coronal sections of the E15 meso-diencephalic dopamine (mdDA) system. (A) TH labels mdDA neurons. (A-C) Whereas no significant Sema7A expression is detected within the area of TH labelling, plexinC1 and TH expression clearly overlaps. (D) The sense control shows no specific labelling. Thus, at E15 plexinC1 but not Sema7A is expressed by mdDA neurons. Scale bar 60 μm (A-D). [file 1471-213X-7-98-S1.pdf]

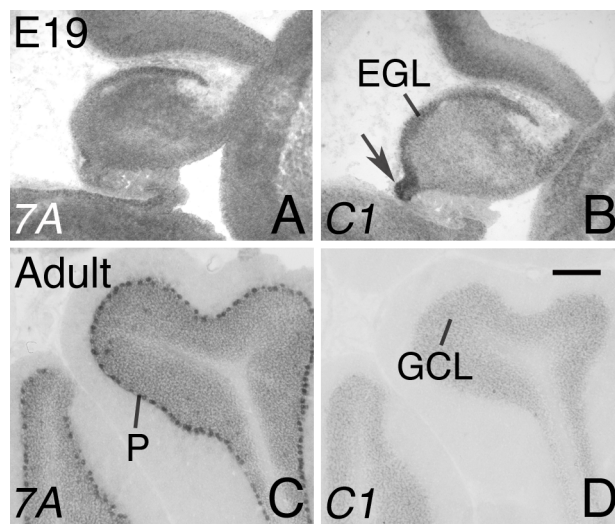

Supplement: Additional file 2 — Sema7A and plexinC1 expression in the embryonic and adult cerebellum. Description: In situ hybridization for Sema7A and plexinC1 on consecutive sagittal sections of the E19 (A, B) and adult cerebellum (C, D). (A, B) Sema7A is widely expressed throughout the developing cerebellum, whereas plexinC1 signals are enriched in the rhombic lip (arrow) and external granule cell layer (EGL). (C, D) In the adult cerebellum, Sema7A is expressed in Purkinje cells (P) and granule cells, whereas plexinC1 only labels few cells in the granule cell layer (GCL). Scale bar 150 μm (A, B), and 245 μm (C, D). [file 1471-213X-7-98-S2.pdf]
